# Expression and prognostic significance of Niemann-Pick C1-Like 1 in colorectal cancer: a retrospective cohort study

**DOI:** 10.1186/s12944-021-01539-0

**Published:** 2021-09-12

**Authors:** Ryuk Jun Kwon, Eun-Ju Park, Sang Yeoup Lee, Youngin Lee, Chungsu Hwang, Choongrak Kim, Young Hye Cho

**Affiliations:** 1 Family Medicine Clinic and Research Institute of Convergence of Biomedical Science and Technology, Pusan National University Yangsan Hospital, Beomeo-ri, Mulgeum-eup, 50612 Yangsan, Gyeongsangnam-do South Korea; 2 Department of Pathology, Pusan National University School of Medicine, 626-780 Yangsan, South Korea; 3 Department of Statistics, Pusan National University, 609-735 Busan, South Korea

**Keywords:** Niemann-Pick C1-Like 1, Ezetimibe, Cholesterol, Colorectal cancer, Prognosis, Overall survival, Stage, Molecular marker

## Abstract

**Background:**

Colorectal cancer (CRC) is a malignancy of the large intestine, whose development and prognosis have been demonstrated to be associated with altered lipid metabolism. High cholesterol intake is associated with an increased risk of CRC, and elevated serum cholesterol levels are known to be correlated with risk of developing CRC. Niemann-Pick C1-Like 1 (NPC1L1), a target of ezetimibe, plays an essential role in the absorption of intestinal cholesterol. However, whether the altered expression of NPC1L1 affects CRC development and prognosis is currently unknown.

**Methods:**

Data corresponding to patients with CRC were obtained from The Cancer Genome Atlas (TCAG). Datasets from the Genome Data Analysis Center (GDAC) platform were analyzed to compare the expression of *NPC1L1* in normal and CRC tissues using the Mann–Whitney U test and chi-square test. Further, the datasets from the Gene Expression Omnibus (GEO) database were analyzed. The log-rank test and multivariate Cox proportional hazard regression analysis were performed to determine whether *NPC1L1* significantly affects the prognosis of CRC.

**Results:**

The expression of *NPC1L1* was found to be upregulated in CRC and was significantly associated with the N and pathological stages but not with the histological type, age, and sex. Increased *NPC1L1* expression in CRC was related to poor patient survival, as evidenced by the Kaplan–Meier and multivariate regression analyses.

**Conclusions:**

As high expression of *NPC1L1* was associated with CRC development, pathological stage, and prognosis, *NPC1L1* can serve as an independent prognostic marker for CRC.

## Background

Colorectal cancer (CRC), a malignancy of the colon and rectum, is the third most common cancer and the fourth leading cause of cancer-related deaths worldwide [1]. The incidence of CRC is rapidly increasing in countries with medium-to-high human development indices, including those in Eastern Europe, South America, and Asia, owing to changes in dietary habits and westernized lifestyles [2, 3]. The incidence of CRC is 19.7 per 100,000 people, and the 5-year survival rate of patients with stage IV or metastatic CRC is 12 % [4]. Age, family history, inflammatory bowel disease (IBD), hereditary CRC, obesity, and diabetes are known risk factors for CRC. Despite the advances in our understanding of CRC pathogenesis, the response of advanced CRC to conventional chemotherapy is poor. Therefore, it is imperative to identify new molecular markers for predicting the prognosis of CRC, thereby improving the survival rates of the afflicted patients.

Many studies have reported that the development and progression of cancers are associated with altered cholesterol metabolism [5]. Oxysterol 27-hydroxycholesterol, a primary metabolite of cholesterol, was found to promote breast cancer progression in mouse models [6], and the level of total cholesterol was positively associated with the incidence of prostate cancer in men [7]. High levels of cholesterol intake are associated with an increased risk of CRC [8], and elevated serum cholesterol levels are correlated with CRC risk [9]. Among the molecules involved in cholesterol metabolism, Niemann-Pick C1-Like 1 (NPC1L1) is a transmembrane protein that is essential for the intestinal absorption of cholesterol [10]. Importantly, NPC1L1 is a target of ezetimibe, a drug that is used to treat dyslipidemia that cannot be managed using statin treatment. NPC1L1 is expressed at high levels in the human small intestine and liver but is expressed at low levels in the colon, kidneys, and brain [11]. NPC1L1 knockdown was reported to result in significantly decreased the number of tumors in a murine model of colitis-associated CRC [12]. However, whether the altered expression of NPC1L1 correlates with the development and prognosis of CRC in humans remains unclear.

In the present study, *NPC1L1* expression in normal and CRC tissues was compared and a correlation was investigated between the expression of *NPC1L1* and clinical characteristics. Moreover, the influence of *NPC1L1* on the overall survival (OS) of CRC patients was examined, and the value of *NPC1L1* as an independent prognostic factor for CRC was determined.

## Methods

### Patients and gene expression data

**Fig. 1:**
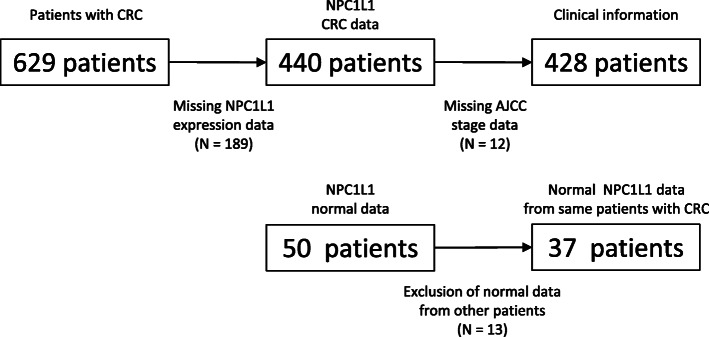
Flow chart for obtaining information for CRC patients with normal *NPC1L1* expression

The Cancer Genome Atlas (TCGA) hosts large clinical datasets with information on DNA methylation, microRNAs and RNA expression in various types of cancers, including CRC. TCGA data corresponding to 629 patients with CRC were obtained as previously described [13]. Among these, data corresponding to 201 patients were excluded from the study because information regarding *NPC1L1* expression for 189 patients and pathological stages for 12 patients was not available. Thus, data corresponding to a total of 428 patients with CRC were finally included for prognosis analysis in this study (Fig. 1). Histological type, age, race, TNM stage, and pathological stage were selected as the clinical variables. However, race as a variable was not eventually used for analysis because no data were available for 36.7 % of the patients whose data were curated in these datasets (Table 1). TNM staging is a system for classifying malignancies, wherein the T stage indicates the size of the primary tumor and the degree of invasion, the N stage indicates the extent to which the tumor has spread to the surrounding lymph nodes, and the M stage indicates the degree of metastasis to other organs. mRNA expression is the read-out of the total number of overlapping reads encompassing a gene during differential expression analysis in RNA sequencing, also known as the read count.

**Table 1:** Clinical characteristics of patients with CRC

Patient characteristics (*N* = 428)		Total (%)
Histological type	Colon adenocarcinoma	363 (84.8)
	Colon mucinous adenocarcinoma	60 (14)
	Unknown	5 (1.2)
Overall survival months (mean ± SD^a^)		29.43 ± 26.08
Age (mean ± SD^a^)		66.63 ± 13.08
Sex	Male	228 (53.3)
	Female	200 (46.7)
Race	American Indian or Alaska native	1 (0.2)
	Asian	11 (2.6)
	Black or American	57 (13.3)
	White	202 (47.2)
	Unknown	157 (36.7)
T stage	Tis	1 (0.2)
	T1-T2	81 (18.9)
	T3-T4	346 (80.8)
M stage	M0	321 (75)
	M1	61 (14.3)
	Unknown	46 (10.7)
N stage	N0	250 (58.4)
	N1-N2	178 (41.6)
AJCC stage	Stage I	72 (16.8)
	Stage II	169 (39.5)
	Stage III	126 (29.4)
	Stage IV	61 (14.3)

^a^*SD* standard deviation, *CRC* colorectal cancer, *AJCC* American Joint Committee on Cancer

Overall, 50 normal datasets comprising data corresponding to *NPC1L1* expression were obtained from the Genome Data Analysis Center (GDAC) platform. Of the 50 normal datasets, 37 datasets containing information regarding *NPC1L1* expression in CRC patients were included in this study. Owing to the fact that the definition of “normal” in the context of expression is based on the level of mRNA expression in the tissues adjacent to the CRC tissues, the expression of *NPC1L1* in normal and CRC tissues from the same patients could be compared. When comparing the relative expression of *NPC1L1* in paired tissues, expression values of *NPC1L1* in each of the 35 tumor tissues were divided by those in paired normal tissues and expressed as the Log_2_ of expression. Two normal datasets were not used because the expression of *NPC1L1* in two tumor tissues (calculated as numerator) was 0.

To confirm the expression and prognostic significance of *NPC1L1* in CRC, *NPC1L1* expression data from the Gene Expression Omnibus (GEO) datasets (GSE9348, GSE17536, and GSE129451) were obtained and analyzed.

### Statistical analysis

The differences between the expression of *NPC1L1* in normal and CRC tissues were examined using the Mann–Whitney U test after subjecting the data to the Shapiro–Wilk normality test as the data were not normally distributed. Statistical significance was set at *P* < 0.05. A chi-square test was performed to analyze the correlation between *NPC1L1* expression and clinical characteristics. Overall survival (OS) was calculated from the date of diagnosis to death or last follow-up. For the Kaplan–Meier (KM) curves, patients with CRC were assigned to either the *NPC1L1* low (*n* = 214) or *NPC1L1* high (*n* = 214) expression group based on the median levels of *NPC1L1* expression. The log-rank test was used to calculate the *P* values. Univariate and multivariate Cox proportional hazard regression analyses were performed to determine whether *NPC1L1* is a significant predictor of OS in patients with CRC. Box and whisker plot construction, KM curve construction, and statistical analysis were performed using the Excel (2016) and SPSS programs (version 20).

## Results

### Baseline characteristics

The characteristics of the patients with CRC are summarized in Table 1. The mean age and OS of patients with CRC were 66.63 ± 13.08 years and 29.43 ± 26.08 months, respectively. Most of the CRC tissues were found to be adenocarcinoma (*n* = 363, 84.8 %), and the ratio between the number of males and females was not notably different (male: *n* = 228, female: *n* = 200). The proportion of Caucasian subjects with CRC was relatively high (*n* = 202, 47.2 %), and the proportion of Asian subjects with CRC was 2.6 %. Based on the American Joint Committee on Cancer (AJCC) staging system, the proportion of patients with stage I, II, III, and IV CRC was found to be 16.8 %, 39.5 %, 29.4 %, and 14.3 %, respectively.

### Expression of *NPC1L1* in normal and CRC tissues

**Fig. 2:**
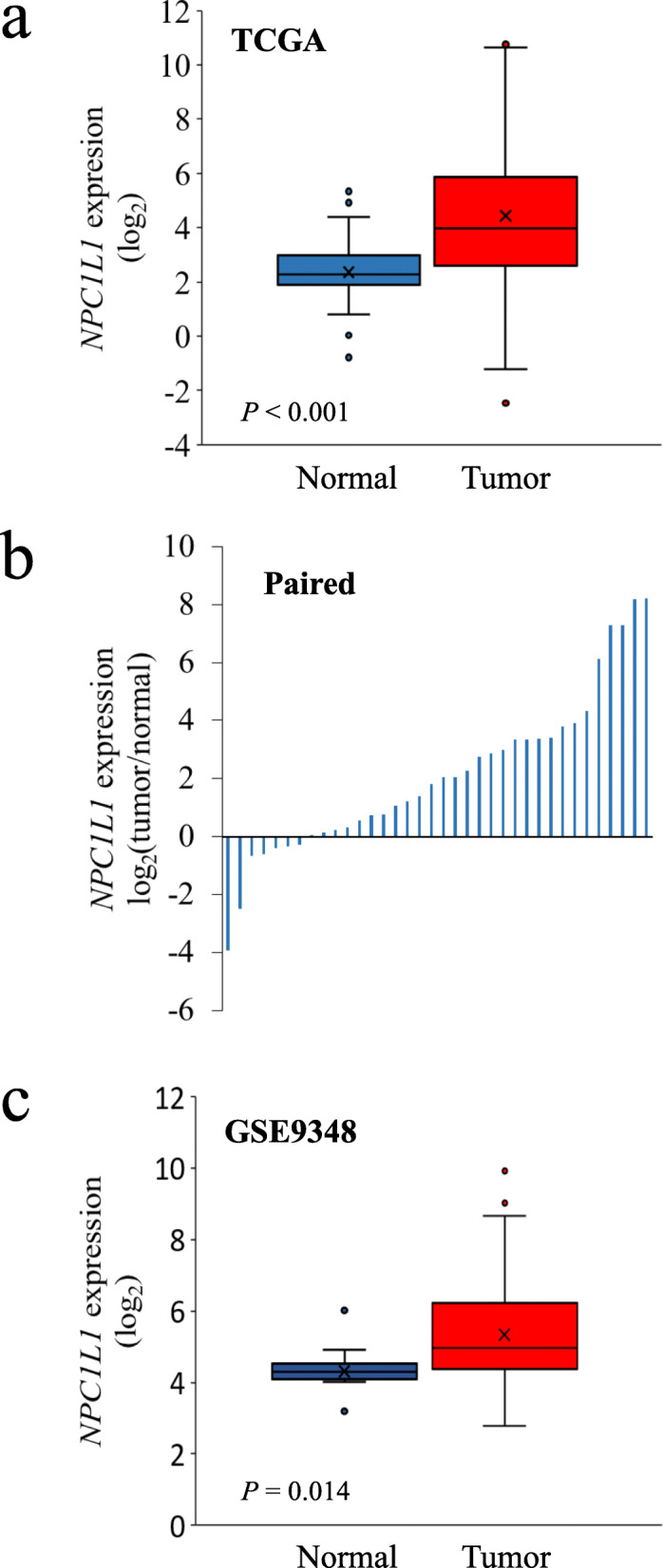
*NPC1L1* expression in normal and CRC tissues. **a** *NPC1L1* expression in CRC tissues as compared to that in normal tissues is presented as a box and whisker plot format. The numbers of normal and tumor tissues analyzed were 50 and 440, respectively. The mean value of *NPC1L1* expression in normal tissues (blue box) was 7.00 and that in CRC tissues (red box) was 130.09. Significance was evaluated using the Mann–Whitney U test (*P* < 0.001). **b** Relative expression levels of *NPC1L1* in tumor tissues (CRC) as compared to those in paired normal tissues are presented as a bar graph. The numbers of tumor and paired normal tissues analyzed were 35 (each). **c** *NPC1L1* expression in CRC tissues as compared to that in normal tissues (GSE9348) is presented as a box and whisker plot format. The numbers of normal and tumor tissues analyzed were 12 and 70, respectively. The mean value of *NPC1L1* expression in normal tissues (blue box) was 22.69 and that in CRC tissues (red box) was 81.35. Significance was evaluated using the Mann–Whitney U test (*P* = 0.014).

*NPC1L1* expression was found to be significantly higher in CRC tissues than that in normal tissues (normal: mean 7.00, CRC: mean 130.09, *P* < 0.05) (Fig. 2a). For accurate verification, the relative expression of *NPC1L1* was analyzed in paired normal and tumor tissues from patients with CRC (Fig. 2b). *NPC1L1* expression was higher in most CRC tissues than that in paired normal tissues. To determine whether the *NPC1L1* expression levels in data—from databases other than TCGA—corresponding to normal and tumor tissues were consistent with the results obtained using TCGA data, a GEO dataset (GSE9348) was analyzed (Fig. 2c). The results indicated that the expression of *NPC1L1* was significantly higher in CRC tissues than that in normal tissues (normal: mean 22.69, CRC: mean 81.35, *P* < 0.05).

### Relationship between *NPC1L1* expression and patient characteristics

Based on the median value of *NPC1L1* expression, the samples were divided into *NPC1L1* low and *NPC1L1* high expression groups and the correlation between *NPC1L1* expression and the patient characteristics was examined (Table 2). The expression level of *NPC1L1* was significantly correlated with the N stage (*P* < 0.05) and pathological stages (*P* < 0.05). However, no correlation was observed between *NPC1L1* expression and the histological type, age, sex, and T and M stages.

**Table 2:** Correlation between *NPC1L1* expression and clinical characteristics of patients with CRC

*NPC1L1* expression				
Characteristic	*N*	Low	High	*P*-value
Histologic type	423			0.257
Adenocarcinoma		186	177	
Mucinous		26	34	
Age (years)	428			0.923
< 70		115	116	
≥ 70		99	98	
Sex	428			0.245
Male		120	108	
Female		94	106	
T stages	428			0.141
Tis, T1–T2		47	35	
T3–T4		167	179	
M stages	382			0.069
M0		167	154	
M1		24	37	
N stages	428			0.006
N0		139	111	
N1–N2		75	103	
AJCC stages	428			0.025
Stage I–II		132	109	
Stage III–IV		82	105	

*AJCC* American Joint Committee on Cancer, *CRC* colorectal cancer, *NPC1L1* Niemann-Pick C1-Like 1

### Five-year OS in the *NPC1L1* low and *NPC1L1* high expression groups

**Fig. 3:**
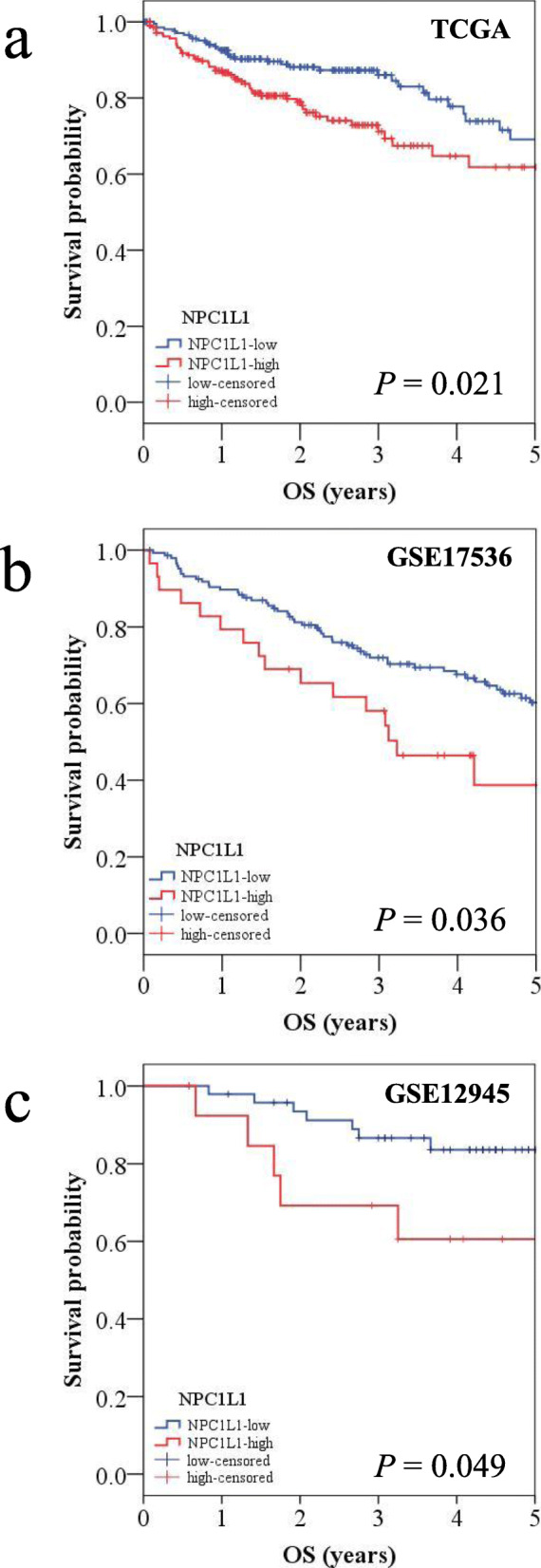
Kaplan–Meier survival analysis based on the expression level of *NPC1L1. ***a** Kaplan–Meier curve based on TCGA dataset. The blue line indicates the group with low *NPC1L1* expression, and the red line indicates the group with high *NPC1L1*expression. The 75th percentiles of low and high *NPC1L1* expression groups were 4.12 and 2.35 years, respectively. The *P* value was calculated using the log-rank method and was significant (*P*< 0.021). **b**, **c** Kaplan–Meier curve based on the GEO dataset (GSE17536 and GSE12945). The cutoff point was obtained using a ROC curve. The blue line indicates the group with low *NPC1L1* expression, and the red line indicates the group with high *NPC1L1* expression. The *P *value was calculated by the log-rank method and was significant (GSE17536, *P *= 0.036; GSE12945, *P* = 0.049)

To determine whether *NPC1L1* expression affects the prognosis of CRC patients, KM analysis was performed for the *NPC1L1* low and *NPC1L1* high expression groups against OS (*NPC1L1* low expression group, *n* = 214; *NPC1L1* high expression group, *n* = 214) (Fig. 3a). Based on the 75th percentile, the OS of the *NPC1L1* low and *NPC1L1* high expression groups was estimated to be 4.1 years and 2.3 years, respectively. Thus, the OS of CRC patients with high NPC1L1 expression was significantly less (*P* = 0.021) than that of CRC patients with low *NPC1L1* expression. To confirm this result, CRC patients whose data were curated in two datasets, i.e., GSE17536 and GSE129451 were first stratified into *NPC1L1* low and *NPC1L1* high groups; 148 and 29 patients whose data were curated in the GSE17536 dataset were classified as *NPC1L1* low and *NPC1L1* high, respectively, whereas 48 and 14 patients whose data were curated in the GSE129451 dataset were classified as *NPC1L1* low and *NPC1L1* high, respectively. Consistent with the results obtained upon analyzing TCGA datasets, CRC patients classified as *NPC1L1* high had significantly poorer survival than those classified as *NPC1L1* low (*P* = 0.036 and 0.049) (Fig. 3b and c).

### Association between *NPC1L1* expression and CRC prognosis

Univariate and multivariate Cox proportional hazards regression analyses were performed to assess the prognostic significance of *NPC1L1* expression (Table 3). In the univariate analysis, age (*P* = 0.015), AJCC stage (*P* < 0.001), and *NPC1L1* (*P* = 0.022) were identified as indicators of OS. Multivariate analysis revealed that age (*P* < 0.001), AJCC stage (*P* < 0.001), and *NPC1L1* (*P* = 0.004) were significantly associated with OS. The hazard ratios for age, stage, and *NPC1L1* were 2.286, 3.698, and 1.618, respectively. The prognostic importance of *NPC1L1* with that of other markers, such as vascular endothelial growth factor A (VEGF-A), metastasis-associated in colon cancer 1 (MACC1), and transforming growth factor-beta 1 (TGF-β1) with respect to CRC was also compared (Table 4). Multivariate regression analysis revealed that the expression of *NPC1L1* (*P* = 0.038) and VEGF-A (*P* = 0.009) was significantly associated with the OS of CRC patients.

**Table 3:** Univariate and multivariate analyses to identify a correlation between prognostic factors and OS in CRC patients

	Univariate analysis		Multivariate analysis	
	HR^a^ (95% CI^b^)	*P*-value	HR^a^ (95% CI^b^)	*P*-value
Age ≥ 70 (*vs.* < 70)	1.669 (1.103–2.524)	0.015	2.286 (1.485–3.519)	< 0.001
Sex Male (*vs.* Female)	0.872 (0.579–1.3174)	0.513	0.942 (0.622–1.426)	0.776
Stage III + IV (*vs.* I + II)	3.150 (2.044–4.855)	< 0.001	3.698 (2.366–5.779)	< 0.001
*NPC1L1* High (*vs.* Low)	1.618 (1.071–2.445)	0.022	1.618 (1.067–2.453)	0.024

^a^HR hazard ratio, ^b^*CI* confidence interval, *OS* overall survival, *CRC* colorectal cancer, *NPC1L1* Niemann-Pick C1-Like 1

**Table 4:** Univariate and multivariate analyses to identify a correlation between NPC1L1 expression and other prognostic factors of CRC

	Univariate analysis		Multivariate analysis	
	HR^a^ (95% CI^b^)	*P*-value	HR^a^ (95% CI^b^)	*P*-value
*NPC1L1* High (*vs.* Low)	1.618 (1.071–2.445)	0.022	1.557 (1.025–2.363)	0.038
*VEGF-A* High (*vs.* Low)	1.796 (1.185–2.723)	0.005	1.758 (1.150–2.686)	0.009
*MACC1* High (*vs.* Low)	1.567 (1.033–2.375)	0.034	1.421 (0.928–2.178)	0.106
*TGF-β1* High (*vs.* Low)	1.369 (0.908–2.064)	0.132	1.502 (0.990–2.278)	0.056

^a^*HR* hazard ratio, ^b^*CI* confidence interval, *CRC* colorectal cancer, *NPC1L1* Niemann-Pick C1-Like 1, *TGF-β1* transforming growth factor beta 1, *MACC1* metastasis-associated in colon cancer 1, *VEGF-A* vascular endothelial growth factor-A

### Survival curves after combining the prognostic factors

**Fig. 4:**
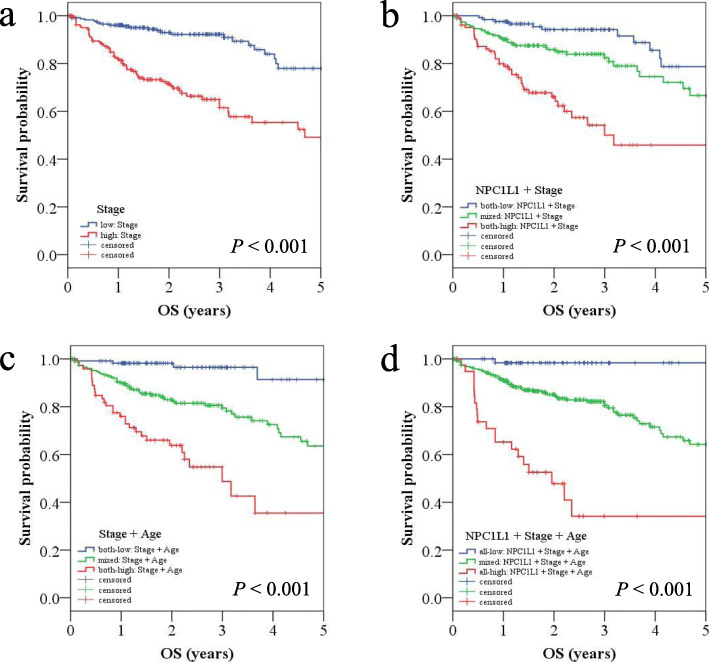
Kaplan–Meier survival analysis after applying a combination of prognostic factors. The stage (low: I + II vs. high: III + IV), age (low: < 70 vs. high: ≥ 70), and *NPC1L1* (low expression vs. high expression) groups were each divided into two groups. Then, survival analysis was performed by combining prognostic factors. The red line indicates a high stage and/or high *NPC1L1* expression and/or older age group. The blue line indicates a low stage and/or low *NPC1L1* expression and/or younger age group. The green line indicates mixed combination groups, excluding all high and all low combination groups. **a** Stage-only group. **b** *NPC1L1* and stage group. **c** Stage and age group. **d** *NPC1L1*, stage, and age group

To identify CRC patients with a clinically worse prognosis, KM plots were generated using a combination of independent factors, such as stage, age, and *NPC1L1* expression. Analysis of data corresponding to patients with CRC low and high stages revealed a median value of 4.68 years for patients with high stage CRC (Fig. 4a). Survival curves generated using a combination of two factors, i.e., *NPC1L1* expression and stage revealed that the median value for CRC patients with the high stage/high *NPC1L1* expression was 3.18 years, and that these patients showed a worse prognosis (Fig. 4b). Next, survival curves were generated using a combination of stage and age; these curves revealed the median value of 3.00 years for patients with high stage/high age, and they also revealed that these patients had a poor prognosis (Fig. 4c). Further, survival curves generated using a combination of three factors, i.e., stage, age, and *NPC1L1* expression revealed that patients with high *NPC1L1*/high stage/high age had the shortest median value among all patients, i.e., 1.96 years (Fig. 4d).

## Discussion

NPC1L1 is essential for the intestinal absorption of cholesterol [10], and is a target of ezetimibe, a drug used to treat dyslipidemia recalcitrant to statin treatment. However, its expression and prognostic relevance in cancers have been poorly characterized. *NPC1L1* expression was found to be downregulated in hepatocellular carcinoma (HCC) [14] but upregulated in pancreatic cancer [15]. In the present study, *NPC1L1* expression was higher in CRC tissues than that in normal tissues (Fig. 2). A previous study revealed an inverse correlation between *NPC1L1* expression and pathological stage, tumor differentiation, and vascular invasion in HCC [14]. In contrast, in the present study, *NPC1L1* expression was positively associated with the N stage and pathological stage (Table 2). This can be explained by the fact that numerous studies have shown that the function of a specific gene in cancer development and progression may differ depending on the cancer type. For instance, increased Notch levels were correlated with tumor grade and metastasis in CRC [16], whereas Notch signaling was found to exhibit tumor-suppressive effects in glioblastoma [17]. Even in the same tumor cells, a gene may modulate cancer progression and suppression in an environment- or time-dependent manner [18, 19]. Thus, the involvement of NPC1L1 in cancer development may be cancer-type specific.

Abnormal lipid metabolism is associated with the development of vascular disorders, hyperlipidemia, lipid storage‒associated diseases, and obesity. Changes in lipid metabolism play an important role in the development and progression of cancer [20–22]. Lipids are used for energy supply, cell membrane structural material, signaling molecules, and post-transcriptional modification of cancer cells ; further, activation of lipogenic genes in cancer cells is known to promote cell proliferation and alter cell characteristics [20]. Studies have confirmed changes in the content and composition of fatty acids, oxylipins, and triacylglycerols in the serum, tumor tissue, and adipose tissue of CRC patients [21]. In addition, the molecules responsible for altered lipid metabolism are thought to serve as potential biomarkers for CRC development, progression, and prognosis [22]. Among them, NPC1L1 is known to be an important molecule for cholesterol absorption and is associated with patient prognosis in HCC [14]. The primary objective of this study was to investigate the difference in the 5-year survival between the *NPC1L1* high and *NPC1L1* low groups because this parameter is widely used to reflect the prognosis—and the endeavors required for the clinical management—of cancer. The results showed that patients in the *NPC1L1* high expression group had poorer OS than those in the *NPC1L1* low expression group (TCGA dataset) (Fig. 3a). Consistent with the results of this analysis, the analysis of datasets from other databases (GSE17536 and GSE129451) also revealed that high *NPC1L1* expression was associated with poor survival outcomes in CRC patients (Fig. 3b and c). Univariate and multivariate analyses revealed that *NPC1L1* expression, along with age and disease stage is significantly correlated with OS in CRC patients (Table 3). To confirm whether *NPC1L1* represents an independent prognostic marker of CRC, its prognostic significance, along with that of other CRC prognostic markers, such as VEGF-A, MACC1, and TGF-β1, was determined using multivariate regression analysis (Table 4). Previous studies have shown that the OS of CRC patients with high expression of VEGF-A or MACC1 is significantly shorter than that of CRC patients with low expression of VEGF-A or MACC1 [23, 24]. A meta-analysis indicated the applicability of TGF-β as a prognostic factor for patients with CRC [25]. In this study, the expression of *NPC1L1*, along with that of VEGF-A, was significantly associated with CRC prognosis. Thus, these results suggest that NPC1L1, along with other known prognostic markers, is an independent prognostic marker for CRC.

Stage and age are recognized as prognostic factors related to mortality and survival in CRC [4]. To identify CRC patients with a worse prognosis in clinical practice based on *NPC1L1* expression, KM plots generated using a combination of prognostic markers (*NPC1L1*, stage, and age) were used (Fig. 4). When a combination of *NPC1L1* expression and stage was used to generate the survival curve, the median value for patients with high *NPC1L1*/high stage was lower than that of patients with high stage alone. Next, when a combination of *NPC1L1* expression and stage/age was used to generate the survival curve, patients with high *NPC1L1*/high stage/high age exhibited a poorer prognosis than those with high stage/high age. Additionally, previous studies have shown that the apoptosis rate was higher in pancreatic cancer cells treated with ezetimibe or si*NPC1L1* than that in control cells; this indicated an efficient method for treating pancreatic cancer [15]. These results suggest that NPC1L1 may be used as a screening marker to individually identify CRC patients with poor prognosis. Importantly, the findings of this study can serve as a basis for investigating the use of ezetimibe as an adjuvant treatment for CRC.

### Strengths and limitations

The present study has several strengths. First, to our knowledge, this is the first study to reveal *NPC1L1* as an independent prognostic factor in CRC. Thus, *NPC1L1* may serve as a marker for enabling the clinical management of CRC patients, thereby improving the survival rates of these patients. Second, the datasets corresponding to data from a large number of CRC patients were used to demonstrate the relationship between high *NPC1L1* expression and poor survival. Third, this study suggests an interesting future research direction for the employment of ezetimibe as an adjuvant treatment for CRC. Despite these strengths, the study has a few limitations. First, information regarding risk factors for CRC, such as family history, smoking habit, obesity, diabetes, and IBD, could not be presented owing to limited data. It may be interesting to identify the relationship between *NPC1L1* expression and risk factors for CRC in the future. Second, no experimental verification for the development and progression of CRC resulting from high *NPC1L1* expression was provided. In particular, the mRNA and protein expression does not always exhibit a proportional relationship. Depending on the gene in question, the mean correlation between mRNA and protein levels may vary from 0.28 to 0.71 [26]. Interestingly, the correlation between the mRNA and protein expression among cancer cell lines (osteosarcoma, epidermoid carcinoma, and glioblastoma) was high, i.e., from 0.58 to 0.63 [27]. Nicolle et al. found that the transcript-level expression of *NPC1L1* was correlated with its protein level in pancreatic cancers [15]. If there is a high correlation between the mRNA and protein levels among cancer cells, the protein expression of NPC1L1 in CRC may also be high in proportion to the expressed *NPC1L1* transcript. To confirm this expectation, further experimental studies are warranted. Although this study does not contribute any experimental evidence *per se*, it provides a framework for further studies aimed at experimentally investigating the role of NPC1L1 at the molecular and cellular levels in CRC.

## Conclusions

This study shows, for the first time, that high *NPC1L1* expression in CRC is associated with low survival. Thus, *NPC1L1* can serve as an independent prognostic marker in CRC patients. Based on the results, clinicians will be able to use *NPC1L1* expression as a marker to identify CRC patients with a poor prognosis. Moreover, the present study demonstrates *NPC1L1* upregulation in CRC and its association with the N stage and pathological stages. These results will contribute toward our understanding of the relationship between CRC and cholesterol and experimentally investigating the role of NPC1L1 in the development and progression of CRC.

## Data Availability

The datasets investigated and analyzed in this study are publicly available.

## Abbreviations

AJCC: American Joint Committee on Cancer
CI: Confidence interval
CRC: Colorectal cancer
GEO: Gene Expression Omnibus
GDAC: Genome Data Analysis Center
HCC: Hepatocellular carcinoma
IBD: Inflammatory bowel disease
KM: Kaplan-Meier
MACC1: Metastasis-associated in colon cancer 1
NPC1L1: Niemann-Pick C1-Like 1
OS: Overall survival
TCGA: The Cancer Genome Atlas
TGF-β1: Transforming growth factor-beta 1
VEGF-A: Vascular endothelial growth factor-A
